# Opportunities for better use of collective action theory in research and governance for invasive species management

**DOI:** 10.1111/cobi.13266

**Published:** 2019-01-04

**Authors:** Sonia Graham, Alexander L. Metcalf, Nicholas Gill, Rebecca Niemiec, Carlo Moreno, Thomas Bach, Victoria Ikutegbe, Lars Hallstrom, Zhao Ma, Alice Lubeck

**Affiliations:** ^1^ School of Social Sciences University of New South Wales Morven Brown Building Room G16 Sydney NSW 2052 Australia; ^2^ W.A. Franke College of Forestry and Conservation University of Montana 440 CHCB, 32 Campus Drive Missoula MT 59812 U.S.A.; ^3^ School of Geography and Sustainable Communities and Australian Centre for Cultural Environmental Research University of Wollongong Northfields Avenue Wollongong NSW 2522 Australia; ^4^ Emmett Interdisciplinary Program in Environment and Resources Stanford University 473 Via Ortega Way, Suite 226 Stanford CA 94305 U.S.A.; ^5^ Environmental Studies Program The College of Wooster Wooster OH 44691 U.S.A.; ^6^ School of Geography The University of Melbourne Parkville VIC 3010 Australia; ^7^ Alberta Centre for Sustainable Rural Communities, Augustana Faculty University of Alberta 4901 46th Avenue Camrose AB T4V2R3 Canada; ^8^ Department of Forestry and Natural Resources Purdue University 195 Marsteller Street West Lafayette IN 47907‐2033 U.S.A.

**Keywords:** alien species, comanagement, cooperation, coordination, non‐native species, participation, social dilemma, co‐manejo, coordinación, cooperación, dilema social, especie invasora, especie no nativa, participación, 外来物种, 非本地物种, 共同管理, 合作, 协调, 社会困境, 参与

## Abstract

Controlling invasive species presents a public‐good dilemma. Although environmental, social, and economic benefits of control accrue to society, costs are borne by only a few individuals and organizations. For decades, policy makers have used incentives and sanctions to encourage or coerce individual actors to contribute to the public good, with limited success. Diverse, subnational efforts to collectively manage invasive plants, insects, and animals provide effective alternatives to traditional command‐and‐control approaches. Despite this work, there has been little systematic evaluation of collective efforts to determine whether there are consistent principles underpinning success. We reviewed 32 studies to identify the extent to which collective‐action theories from related agricultural and environmental fields explain collaborative invasive species management approaches; describe and differentiate emergent invasive species collective‐action efforts; and provide guidance on how to enable more collaborative approaches to invasive species management. We identified 4 types of collective action aimed at invasive species—externally led, community led, comanaged, and organizational coalitions—that provide blueprints for future invasive species management. Existing collective‐action theories could explain the importance attributed to developing shared knowledge of the social‐ecological system and the need for social capital. Yet, collection action on invasive species requires different types of monitoring, sanctions, and boundary definitions. We argue that future government policies can benefit from establishing flexible boundaries that encourage social learning and enable colocated individuals and organizations to identify common goals, pool resources, and coordinate efforts.

## Abstract 2

*Article impact statement*: Invasive species control requires collective actions that prioritize flexible boundaries and social learning over monitoring and sanctions.

## Abstract (es)

Oportunidades para un Mejor Uso de la Teoría de Acción Colectiva en la Investigación y el Control del Manejo de Especies Invasoras

**Resumen**

El control de especies invasoras es un dilema para el bien público. Aunque los beneficios ambientales, sociales y económicos del control se acoplan a la sociedad, los costos los resuelven unos cuantos individuos y organizaciones. Durante décadas, los legisladores han utilizado incentivos y sanciones para alentar y forzar a los actores individuales a contribuir con el bien público, aunque con un éxito limitado. Los diversos esfuerzos sub‐nacionales para manejar colectivamente plantas, insectos y otros animales proporcionan alternativas efectivas a las estratégicas tradicionales de comando y control. A pesar de esta labor, ha habido una evaluación sistemática mínima de los esfuerzos colectivos para determinar si hay principios congruentes que estén respaldando el éxito. Revisamos 32 estudios para identificar la extensión a la cual las teorías de acción colectiva a partir de los campos agrícolas y ambientales relacionados explican las estrategias colaborativas de manejo de especies invasoras; describen y diferencian los esfuerzos de acción colectiva para especies invasoras emergentes; y proporcionan una guía sobre cómo posibilitar estrategias más colaborativas para el manejo de especies invasoras. Identificamos cuatro tipos de acción colectiva enfocada en las especies invasoras – guiada externamente, guiada por la comunidad, co‐manejada, y coaliciones organizacionales – que proporcionan los planos para el manejo futuro de especies invasoras. Las teorías existentes de acción colectiva podrían explicar la importancia que se le atribuye al conocimiento compartido en desarrollo sobre los sistemas socio‐ecológicos y la necesidad de capital social. Aun así, la acción colectiva sobre las especies invasoras requiere de diferentes tipos de monitoreo, sanciones y definición de los límites. Argumentamos que las próximas políticas gubernamentales pueden beneficiarse del establecimiento de límites flexibles que alientan el aprendizaje social y permiten que individuos y organizaciones colocadas identifiquen objetivos en común, acumulen y compartan recursos y coordinen esfuerzos.

## 摘要

入侵物种的控制是公共利益面临的一个困境。虽然入侵物种控制给环境、社会和经济带来的收益由社会共享, 但支出却只由少数个人和组织承担。几十年来, 决策者用激励和制裁的方式鼓励或迫使个人为公共利益做出贡献, 但成效有限。有许多地方性的工作致力于共同管理入侵植物、昆虫和动物, 为传统的指挥控制型方法提供了有效替代。尽管这样的工作已经展开, 然而却很少有对这些集体努力的系统评估, 来确定是否有统一的原则奠定成功的基础。我们综述了 32 项研究以确定相关农业和环境领域中的集体行动理论在多大程度上可以解释入侵物种的协作管理方法, 描述和区分新出现的入侵物种管理的集体行动, 并对如何促进合作来管理入侵物种提供了指导。我们划分了四类针对入侵物种的集体行动, 分别是外部领导、社区领导、共同管理和组织联盟, 这为未来的入侵物种管理提供了蓝图。现有的集体行动理论可以解释发展社会生态系统共识的重要性及对社会资本的需求。然而, 对入侵物种采取的集体行动需要不同类型的监督、制裁和范围界定。我们认为未来的政府政策可以受益于建立灵活的范围来促进社会学习, 帮助位于同一个地区的个人和组织确定共同目标、汇集资源并协调努力。**【翻译: 胡怡思; 审校: 聂永刚】**

## Introduction

Invasive species are mobile, have multiple vectors, and ignore property, jurisdictional, and tenure boundaries. They are a globally persistent and growing problem for agricultural, forestry, aquatic, and natural systems (Pimental et al. 2001; McGeoch et al. 2010). Economically, invasive species cause considerable losses to agriculture, forestry, aquaculture, and livestock, and management costs are high (Simberloff et al. 2005). Ecologically, invasive species, second only to habitat loss, drive species extinction (CBD 2013). Socially, invasive species threaten ecosystem services and human well‐being (Pejchar & Mooney 2009). Researchers argue that these impacts persist because invasive species management has focused largely on individual‐property solutions at the expense of collaborative approaches that transcend ownership and jurisdictional boundaries in a variety of social‐ecological systems (Epanchin‐Niell et al. 2010; Graham 2013; Ervin & Frisvold 2016). We synthesized existing empirical research on invasive species collective action to enhance understanding of how collaborative approaches work in practice and to determine to what extent they are consistent with, or go beyond, existing collective‐action theories and what lessons can be learned to facilitate cooperative management of invasive species.

Command‐and‐control legislation has formed the foundation of invasive species policies in countries such as Australia (Parsons & Cuthbertson 2001), Canada (Zanden et al. 2010), and the United States (Zellmer 2000), despite the promise of alternative strategies (Head et al. 2015). This approach is predicated on the idea that incentives and sanctions are required to make controlling invasive species the rational choice for individual actors. Recently, policy makers have acknowledged that this approach has not, and cannot, achieve landscape‐scale invasive species control (NSW NRC 2014, 2016). Thus, new approaches are needed that embrace shared responsibility and encourage collective action across land tenures (e.g., NSW NRC 2014, 2016; Great Britain Non‐Native Species Secretariat 2015).

**Figure 1:**
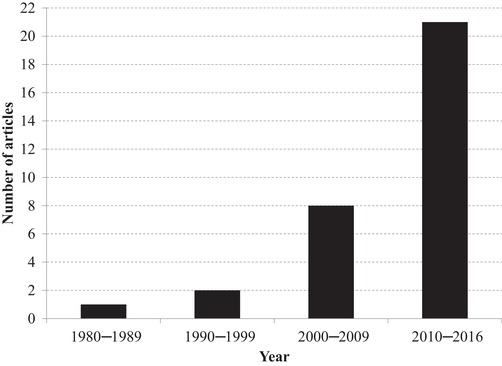
Years of publication of 32 empirical research articles on local and regional collective action in invasive species management.

Theories about collective action have evolved substantially from purely rational explanations (Olson 1965) to more bounded approaches recognizing the importance of other factors, such as the number and heterogeneity of participants, face‐to‐face communication, trust, reputation, and reciprocity (Van Vugt & Snyder 2002; Ostrom 2010). Research into the extent to which collective action exists in invasive species management at subnational scales and the constraints and opportunities to area‐wide cooperation is growing (Fig. 1). Yet, theoretical questions remain about how contemporary collective‐action theories apply to the specifics of invasive species management (Epanchin‐Niell et al. 2010; Niemiec et al. 2016).

Coupling current policy directions with salient lessons from the bounded‐rationality approach and the 30‐year history of invasive species collective action research (beginning with Tette et al. 1987) (Fig. 1) offers an opportunity to provide a critical review of what has been learned, suggestions for more effective application, and prioritization of future research investments. We sought to identify the extent to which collective‐action theories from related agricultural and environmental fields explain collaborative invasive species management approaches; describe and differentiate emergent invasive species collective‐action efforts; and provide practical guidance on how future research, policy, and practice can investigate and facilitate more collaborative approaches. Based on our review, we summarized the theoretical frameworks used to design and assess invasive species collective actions and devised a typology of collective responses to invasive species threats. We considered opportunities to enhance theory and research on invasive species public goods (PGs) and their application across scales.

## Need for Conceptual Clarity

Defining collective action is deceptively difficult. Meinzen‐Dick et al. (2004:200) suggest that, “what most definitions have in common is that collective action requires the *involvement of a group of people*, it requires a *shared interest* within the group, and it involves some kind of *common action* that works in pursuit of that shared interest.” Collective‐action problems can be conceptualized as either common pool resources (CPRs) or PG dilemmas. Both types of resources can be nonexcludable, meaning people cannot be prevented from enjoying them. All PGs are nonexcludable, whereas only subsets of CPRs are nonexcludable (Ostrom 1990). A CPR is rivalrous such that consumption reduces availability for others, whereas PGs are nonrivalrous (Olson 1965; Ostrom 1990; Kollock 1998). Further, CPR dilemmas require restraints on consumption, whereas PG dilemmas do not. Conversely, PG dilemmas require contributions from people (Van Vugt & Snyder 2002), whereas this is true of only a subset of CPR dilemmas (Ostrom 1990; Bisaro & Hinkel 2016). Despite the theoretical clarity between PGs and CPRs, their boundaries can be blurry. Marine resources are often cited as a classic CPR because it is difficult to exclude users, even though a fish harvested by one person cannot be caught by others (Ostrom 1990). National defense is generally considered a PG because protection enjoyed by one does not limit the protection enjoyed by others (Apesteguia & Maier‐Rigaud 2006).

There is widespread agreement that management of invasive species constitutes a collective‐action problem (Epanchin‐Niell et al. 2010; McLeod & Saunders 2011; Yung et al. 2015). The challenges associated with promoting collective actions to manage invasive species are a social dilemma that arises because invasive species freely cross property boundaries, creating uncompensated interdependencies and externalities among spatially proximate land managers (Cornes & Sandler 1996). Yet, there is disagreement about which type of collective‐action problem invasive species management constitutes. Although some authors treat it as a CPR dilemma (e.g., Ervin & Jussaume 2014; Kruger 2016a) or argue it has elements of both CPR and PG dilemmas (e.g., Ervin & Frisvold 2016), more authors consider it a PG problem (e.g., Ayer 1997; Perrings et al. 2002; Toleubayev et al. 2007; Coutts et al. 2013; Graham 2014).

We assert that invasive species control has 2 characteristics that are more consistent with PG dilemmas. First, invasive species control requires contributions (e.g., adopting control practices and supporting local programs) by actors in a system (acknowledged by Kruger [2016a] but was not considered when she categorized invasive species control as a CPR problem). In some cases, restraint is also required. For example, managing herbicide‐resistant weeds requires land managers limit (restrain) their use of some herbicides (Ervin & Jussaume 2014; Ervin & Frisvold 2016). However, restraint cannot solve the overarching problem of invasive species management; contributions are required to remove invasive species. Second, invasive species management generates environments free of invasive species, which are inherently nonrivalrous (Kruger 2016a); one's enjoyment of a weed‐free environment does not detract from another's enjoyment thereof (although noncontributors affect the benefits contributors receive [Kruger 2016a]). The distinctions between PG and CPR problems rest on whether contributions or restraint are required and whether benefits are subtractable (i.e., rivalrous). Although such distinctions can sometimes be nuanced, they define contexts in which human behaviors can vary substantially (Kollock 1998; Bisaro & Hinkel 2016).

Still, investigations of environmental collective action draw primarily from CPR, rather than PG, theories. From Hardin's (1968) “tragedy of the commons” and its subsequent critiques, dialogue in the conservation community has primarily focused on CPRs. Specifically, Ostrom's (1990) 8 design principles for community‐based programs, derived from examples of long‐enduring CPR institutions, have had profound influence on the field. Similarly, Ostrom's (2009) 10 factors that affect self‐organized collective action stem from CPR examples and have been employed in countless studies of environmental collective action. Meta‐analyses of CPR institutions demonstrate that each design principle is individually correlated with successful CPR institutions (Cox et al. 2010) and that some subsets result in more successful CPR institutions, depending on mobility of the resource and amount of human effort required to manage it (Baggio et al. 2016). Although governance arrangements that are most effective for CPRs are likely to differ from PGs (Cox et al. 2010), there has been little systematic consideration of which design principles are applicable to PGs and under what circumstances. In the case of invasive species management, CPR‐focused conceptual models have been applied to investigations of invasive species collective action with little consideration of whether they are appropriate given that invasive species control is more consistent with PG dynamics.

We sought to improve the science and practice of invasive species control by describing ways collective action has manifested and providing theoretical clarity by evaluating the appropriateness of applying a CPR frame to this PG dilemma. We evaluated whether Ostrom's 2 conceptual models, originally developed through study of CPRs, improve understanding of invasive species PG problems. We also considered whether there are additional factors that supplement Ostrom's models and help explain collective action for invasive species control.

## Overview of Invasive Species Collective‐Action Research

Seven of us met and discussed our understanding of collective action in the context of invasive species management. Our interpretation of collective action was oriented toward subnational strategies that encourage coordination, cooperation, and joint action, rather than concurrent unilateral action (consistent with Sadoff and Grey's [2005] cooperation continuum). Our interpretation of invasive species included invasive plants, animals, and insects. We searched for articles that discussed pest management because the terms *pest* and *invasive species* are often used interchangeably (e.g., Epanchin‐Niell et al. 2010; Ford‐Thompson et al. 2012) and because the mobility of pests also requires collective solutions (Ervin & Frisvold 2016).

We identified 21 publications (Supporting Information) related to collective action for invasive species control. We reviewed these publications to refine our conceptualization of collective action and develop a list of search terms (Table 1). Subsequently, we conducted a Scopus title‐abstract‐keyword search on 11 October 2016 and found 144 articles, from which we reviewed those that reported social research about collective invasive species management. Selected articles (32) represented environmental, agricultural, biological, and social sciences and multidisciplinary fields.

**Table 1:** Search terms used to identify articles on collective action in invasive species management

Collective action	Invasive species management
“*collective action*” OR “cooperative control” OR “community reciprocity” OR “community participation” OR “centrally organized” OR “community problem solving” OR “community‐based conservation” OR “community‐based natural resource management” OR “collective natural resource management” OR “community‐based comanagement” OR “social dilemma”	*invasives* OR “*invasive species*” OR “alien species” OR “non‐native species” OR “non‐indigenous species” OR “exotic species” OR “introduced species” OR “biological invasions” OR “biological invasion” OR bioinvasion^*^ OR “invasive animal” OR “invasive animals” OR “exotic animal” OR “exotic animals” OR “feral animal” OR “feral animals” OR “non‐native mammal” OR “non‐native mammals” OR “introduced mammal” OR “introduced mammals” OR feral^*^ OR “invasive plant” OR “invasive plants” OR pest^*^ OR weed^*^ OR biosecurity OR “noxious plant” OR "noxious plants”

We reviewed all articles for general characteristics, such as geographic focus and invasive species of interest, and to determine which PG and CPR theories, if any, were used by authors to frame investigations. Two authors independently coded each article to determine whether Ostrom's 10 factors or 8 design principles were explicitly or implicitly identified as significant to the achievement of collective action for invasive species control, consistent to Cox et al.’s (2010) and Baggio et al.’s (2016) approach. We also identified instances where CPR approaches were inconsistent with the PG nature of invasive species control. We agreed that a singular summary of invasive species collective action was inadequate because of the diverse ways actors organized themselves and that a typology was more appropriate. Using Uetake's (2013) typology as a starting point, we revisited articles to extract descriptions for 4 types of invasive species collective control.

### General Characteristics

The majority of the 32 articles focused on invasive species management in the United States and Australia (Supporting Information). Two studies included comparisons between the United States and Canada and Mexico. Four studies focused exclusively on developing countries. One article focused on continents rather than countries.

### Conceptualizing Invasive Species Management as Common Pool Resources or Public Goods

Articles presented divergent theoretical conceptualizations of invasive species management as a CPR, PG, or nonspecific type of collective‐action problem. Seventeen studies labeled invasive species as a generic collective action problem, 4 considered invasive species a PG problem (Ayer 1997; Toleubayev et al. 2007; Graham 2014; Niemiec et al. 2016), 1 referred to invasive species as a CPR problem (Kruger 2016a), and 1 acknowledged that herbicide susceptibility has both PG and CPR elements (Ervin & Frisvold 2016). The remaining 9 studies did not explicitly frame invasive species with collective action theory or language, despite discussion of cross‐boundary issues or collective responses.

A small subset of CPR factors (Ostrom 2010) and design principles (Ostrom 2009) were mentioned consistently across articles (Tables 2 & 3). The 3 factors most commonly mentioned were number of users, norms or social capital, and knowledge of the socioecological system. The 3 design principles most consistently referenced were monitoring, graduated sanctions, and clearly defined boundaries. Despite this, there were important differences between how these factors and design principles were originally conceptualized in the CPR literature and how authors applied them.

**Table 2:** Results from coding each article against Ostrom's (1990) design principles illustrated by long‐enduring common pool resource institutions.^a^

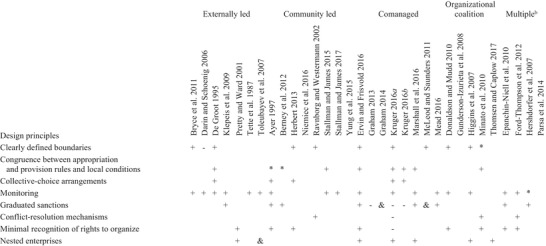

a Codes: blank cell, design principle not identified. In the remaining cells, the design principle was identified: +, behaved as per Ostrom; –, did not behave as per Ostrom; &, did and did not behave as per Ostrom; *, insufficient information to ascertain how the design principle operated.

b Articles spanned multiple forms of collective action.

**Table 3:** Results from coding each article against Ostrom's (2009) 10 factors that affect self‐organized collective action.^a^

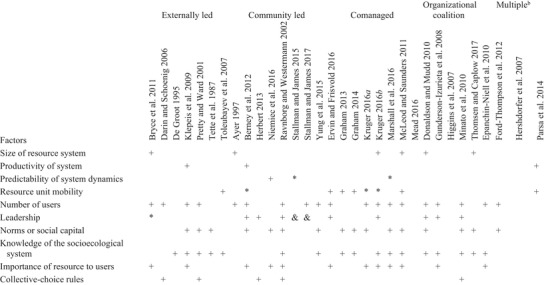

a Codes: Blank cell, design principle not identified. In the remaining cells, the design principle was identified: +, behaved as per Ostrom; &, did and did not behave as per Ostrom; ^*^, insufficient information to ascertain how the design principle operated.

Articles spanned multiple forms of collective action.

Some aspects of CPR theory referenced consistently across articles are applicable to both CPR and PG problems. For example, reviewed articles emphasized the importance of shared problem framing by stakeholders and land managers. Common understanding of the social and ecological contexts and threats posed by invasive species was presented as important for establishing a shared vision and realistic goals (Thomsen & Caplow 2017). Thus, many authors concluded that sharing information and social learning were necessary factors for addressing invasive species problems (e.g., Ayer 1997; Toleubayev et al. 2007; Mead 2016). Trust and reciprocity were frequently cited as necessary ingredients for effective collective responses to invasive species. Consistent with CPR theory, authors emphasized that trust was important for facilitating communication (Graham 2013), developing a sense of community responsibility (Yung et al. 2015; Marshall et al. 2016), and social norms (Minato et al. 2010). Limits to norms and trust were also recognized. For example, norms facilitate mutual aid, but not other forms of collaboration (Niemiec et al. 2016), and trust between private land managers and government staff can undermine collaboration among private land managers (Graham 2013).

Other identified factors reflected CPR theory but had some features that highlighted the PG nature of invasive species management problems. For instance, many articles discussed the difficulty of coordinating a large and diverse population of land managers, reminiscent of the “number of users” factor established by Ostrom (2009) as important for CPRs (e.g., Klepeis et al. 2009; McLeod & Saunders 2011; Yung et al. 2015). Although PG and CPR problems become more difficult with increasing numbers of people involved, the reasons self‐organization becomes harder differ. With PG problems, there are increased transaction costs associated with organizing large groups (Ostrom 2009), but the likelihood of defectors is higher in large groups and is particularly problematic in weakest link PG problems, where invasive species control depends on contributions made by the least (weakest link) willing actors (Hirshleifer 1983). More contributions are necessary to attain the PG (especially in threshold‐aggregator problems, where benefits do not accrue until enough contributions are made), and the failure of some individuals to sufficiently contribute may undermine cooperation through diminished perceptions of efficacy (Kollock 1998).

There are also important differences in the significance attached to boundary definition. Boundaries around CPRs are important for delineating resources, jurisdictional authority, and access rights for users and nonusers. For example, institutions that clearly define users and boundaries are more likely to be successful (Cox et al. 2010). Similarly, having clearly defined users is a necessary condition for success, especially for highly mobile resources (Baggio et al. 2016). This differs from how boundaries related to collective responses to invasive species control where they inspired cooperation, established a scale for the problem that seemed surmountable to local actors, aligned with political boundaries, or leveraged resources (Higgins et al. 2007; Donaldson & Mudd 2010). For example, unclear boundaries of weed management areas represent a missed opportunity to use boundaries to inspire cooperation and solidarity (Gunderson‐Izurieta et al. 2008). Sometimes boundaries are presented as fuzzy (e.g., Ervin & Frisvold 2016) and expandable (e.g., Higgins et al. 2007; Bryce et al. 2011). Although CPR boundaries often have a gradient quality to them, or are fuzzier than the design principles imply (Cox et al. 2010), there has been little consideration of how expandable boundaries can be used to motivate greater participation in collective action.

Monitoring and sanctions by regulators, 2 design principles that lead to more successful CPR institutions (Cox et al. 2010), were sometimes incompatible with the PG nature of invasive species problems and undermined collective efforts. Sustainable use of CPRs is ensured through careful (and collaborative) governance of consumption. For these resources, monitoring of user behavior and the resource conditions by regulators accountable to the users and associated graduated sanctions are essential for detecting, preventing, and punishing unauthorized behavior (Cox et al. 2010). In contrast, PGs are produced through contributions. Sanctions can coerce contributions, but may undermine cooperation when access for monitoring is not ubiquitously available. We found that monitoring and sanctioning of individual behavior by external agencies was onerous (Ervin & Frisvold 2016), undermined cooperation within invasive species programs, and eroded trust among invasive species control advocates and administrators (Hershdorfer et al. 2007; Marshall et al. 2016), especially when monitors were not accountable to the PG contributors (Graham 2013, 2014). Conversely, monitoring of the overall invasive species abundance or distribution was critical to the success of some invasive species control programs (e.g., Tette et al. 1987; de Groot 1995; Hershdorfer et al. 2007). In meta‐analyses of CPR studies, external monitoring of user behavior and the resource condition are lumped together. Our review suggests that for PGs, these 2 forms of monitoring have different impacts on collective action. Furthermore, we found that neighbor‐to‐neighbor, as distinct from external, monitoring motivated contributions to the PG (e.g., Graham 2014; Niemiec et al. 2016), especially in community‐led collective action. More research is needed to understand how different types of monitoring and sanctions (i.e., behavior vs. resource condition and external vs. neighbor‐to‐neighbor) affect contributions to different types of invasive species collective action and other PGs more broadly.

### Four Types of Invasive Species Collective Action

Theoretical inconsistency was further complicated by diverse descriptions of collective‐action approaches to invasive species control. Authors considered 4 types of collective action: externally led, community led, comanaged, and organizational (summarized in Table 4). Differences between the types were not always clear because articles lacked adequate information and because of the diversity of deployment strategies.

**Table 4:** Four types of collective action identified in the 32 articles reviewed

Type	Definition	Examples of individuals or organizations leading collective efforts
Externally led	External agencies or organizations envision, champion, and fund efforts to promote widespread contributions to invasive species control. Such efforts typically include financial incentives or penalties or technical support to landowners.	national, state, or local governments, international nongovernmental organizations (NGOs), state‐sponsored extension programs, or university research teams
Community led	Private landowners or residents provide support, apply social pressure, or organize collaborative efforts with other landowners to control invasive species across property boundaries.	private landowners or residents
Comanaged	Private landowners or residents enter in cooperative arrangements with external agencies or organizations to promote invasive species control at a landscape scale. External agencies or organizations often provide regulations and litigation, incentives, technical assistance, or educational outreach.	agencies or organizations (e.g., state and local government agencies, private companies, NGOs, and universities) and private landowners and residents
Organizational coalitions	Institutions with a formal or informal mesolevel authority and formal networks of government agencies cooperate to control invasive species at a regional scale. Such organizational coalitions coordinate invasive species management programs and activities, pool resources, encourage consistent regulation and engagement, or facilitate management at appropriate ecological scales.	organizations such as Cooperative Weed Management Areas or Cooperative Invasive Species Management Areas in the United States (i.e., partnerships of local, state, and federal government agencies, private landowners, interested stakeholders, and organizations with environmental mandates)

Externally led collective actions were envisioned, championed, and funded by agencies or organizations, such as national, state, or local governments, international nongovernment organizations (NGOs), state‐sponsored extension programs, or university research teams. These entities recognized the cross‐boundary nature of invasive species and invested in landscape‐wide solutions. External groups most often promoted the collective interest by encouraging individual actions toward a PG. Although in 1 case, a large centralized government agency singlehandedly provided the PG (Toleubayev et al. 2007). This type of collective action strongly resembles the external agency‐led type described by Uetake (2013), except that it often sought to organize rural land managers in general (Klepeis et al. 2009), rather than just farmers, and occasionally involved publically and privately managed land of high conservation value (Higgins et al. 2007).

The frequent aim of externally led collective action was to facilitate individual land managers’ access to technology and assistance for addressing species invasions. The high capacity and institutional support of these organizations afforded actions and investments generally unavailable otherwise. Many developed unique science‐based solutions (e.g., universities), provided direct funding to land managers (e.g., state agencies), or facilitated information sharing (Tette et al. 1987; Darin & Schoenig 2006; Bryce et al. 2011). However, with a reliance on external funding and leadership, this type of collective action rarely persisted after outside investments ended (Tette et al. 1987; De Groot 1995; Darin & Schoenig 2006; Toleubayev et al. 2007).

Because externally led collective action largely encouraged independent, rather than collaborative or joint, actions (Sadoff & Grey 2005), none of the articles pertaining to this form of collective action mentioned the need for conflict‐resolution mechanisms (Table 2) or predictable dynamics of the socioecological system (Table 3). Instead, the emphasis was on monitoring to identify new invasions and expanding project boundaries to involve more individuals.

Externally led collective action sought cross‐boundary solutions to invasive species by emphasizing best management practices among independent actors. There is strength in this approach because collaboration requires investments that may overwhelm individual land managers and outside entities can provide landscape‐scale perspectives on emergent threats. However, inspiring local ownership of the problem and solution (Lachapelle & McCool 2005) may help realize the benefits of external investment while ensuring program sustainability. For example, programs might encourage individual responses by local actors while enlisting local participation in program leadership to build consensus around the need for cross‐boundary cooperation. Further, programs could solicit feedback and develop local capacity and revenue to ensure program viability beyond the loss of external support.

Community‐led collective action typically involved private landowners, residents, and sometimes public land managers collaborating to control invaders, often without government mandates or leadership. This form of collective action arose from a shared understanding among landowners that invasive species posed a collective threat requiring coordinated management (Berney et al. 2012; Stallman & James 2017). Such community‐led collective action was observed in rural (Ravnborg & Westermann 2002; Herbert 2013), peri‐urban (Niemiec et al. 2016), and agricultural (Ayer 1997) landscapes. It took diverse forms, including neighbors sharing information and control strategies (Herbert 2013; Niemiec et al. 2016), residents convincing neighbors to or helping them control invasive species on their property (Ravnborg & Westermann 2002; Herbert 2013; Yung et al. 2015; Niemiec et al. 2016), cooperative scouting for pest outbreaks (Stallman & James 2015), coordinated pesticide application, release of biological controls, and crop rotation across farms (Ayer 1997; Stallman & James 2015).

This type of collective action is consistent with Uetake's (2013) nonorganization style because community members collaborate without external involvement. However, some land managers formed independent organizations (e.g., Herbert 2013) and the norms and social capital factor were no more important for this than the other types of collective action (Table 3). No articles mentioned the need for daily communication (Uetake 2013). Further, shared knowledge of the socioecological system was less likely to be important (Table 3). This indicates community‐led collective action starts when there is agreement that something needs to be done, but ambiguity exists regarding who should make decisions or what constitutes appropriate action (Brugnach et al. 2011). For example, in 1 case, landowners started cooperating once they began talking about the impacts inaction was having on each other, rather than taking a systems perspective (Ravnborg & Westermann 2002).

Almost all CPR factors and design principles were mentioned in the descriptions of community‐led collective action (Tables 2 & 3). Yet, there was little overlap in the sets of factors or principles identified in each article, which made it difficult to determine whether any were consistently important. Some factors supported or undermined community‐led collective action, depending on the social context. For example, strong social norms and perceived reciprocity among neighbors regarding invasive species control efforts sometimes enabled collective action (Niemiec et al. 2016), whereas norms in rural areas against approaching neighbors about land management decisions undermined cooperation (Ravnborg & Westermann 2002).

The articles reviewed suggested diverse strategies that can facilitate community‐led collective action. Several studies highlighted the importance of community workshops and organizations that facilitated socialization among community members, information and resource sharing, and informal agreements about invasive species control behavior (Herbert 2013; Niemiec et al. 2016). This was particularly important in places with high neighborhood turnover and absentee landowners (Berney et al. 2012; Yung et al. 2015). To help overcome existing norms that militate against cooperation, organizations and community leaders can highlight the transboundary nature of invasive species control and the ecological and economic benefits that can arise from cooperation among neighbors (Ravnborg & Westermann 2002).

Cooperative arrangements between institutional actors and land managers provided an alternative approach for area‐wide invasive species management. Although Uetake (2013) considers this approach a combination of the externally led and community‐led types, we argue it goes beyond the other 2 approaches because it often involves polycentric governance. In this type, organizational actors included state and local government agencies, private companies, NGOs, and universities, whereas individual actors were typically private landowners. Cooperation could be induced or motivated through diverse mechanisms, such as regulations and litigation (Graham 2013, 2014), incentives (Ervin & Frisvold 2016), technical assistance and educational outreach (Kruger 2016a, 2016b), and community‐based approaches (Graham 2013, 2014).

Two features of comanaged collective action that set it apart from self‐organizing CPR arrangements were how actors took advantage of resource‐unit mobility to galvanize action and how a diversity of users were embraced. For example, recognition of the ease with which invasive species move across the landscape was used to unite organizations and individuals around a common goal (McLeod & Saunders 2011; Graham 2013; Ervin & Frisvold 2016) and instill a sense of community responsibility for managing invasive species (Mead 2016). Outreach and training that embraced diverse actors served to promote trust, communication, and opportunities for social learning and action (Mead 2016).

One of the key challenges for this model of collective action arose from the use of monitoring and sanctioning of landholder behavior (Table 2). Articles that discussed this type of collective action often focused on the weakest link nature of invasive species management. Land managers who participate in comanaged collective action often do so with the expectation that government staff will use their enforcement powers to identify, work with, and sanction those actors who are least prepared or willing to manage invasive species (Graham 2013, 2014). Given that regulations and enforcement tend to erode trust and cooperation (McLeod & Saunders 2011; Marshall et al. 2016), it is challenging to work with unprepared or unwilling land managers in a comanaged collective action framework.

Policy makers and practitioners who wish to encourage comanaged collective action should consider prioritizing community‐building activities and learning opportunities that build trust among land managers and external actors; demonstrate how resource mobilities create interdependencies among diverse actors and the need for a common goal; and encourage increased participation by highlighting positive experiences of participants and emphasizing multiple forms of incentive (financial and nonfinancial) over enforcement of sanctions. Further, investing in invasive species management research that is interdisciplinary and participatory can foster fair representation and shared decision making among stakeholders and lead to socially acceptable and accessible strategies.

Organizational coalition collective action included the regional rearrangement of institutions with a formal or informal meso‐level authority (Donaldson & Mudd 2010), as well as formal networks of government agencies and other organizational actors (Gunderson‐Izurieta et al. 2008; Thomsen & Caplow 2017) that aimed to address invasive species concerns at a regional scale. These included organizations such as Cooperative Weed Management Areas or Cooperative Invasive Species Management Areas in the United States, which were partnerships of local, state, and federal government agencies, private landowners, and various interested groups with a role in invasive species management.

The coalitions ranged from administrative or bureaucratic actions (e.g., networks of functionally or administratively similar offices, centers, or agencies) to community‐based and informal collaborations. In turn, collective action varied from highly formalized, jurisdictionally specialized coordination to informal initiatives that emphasized shared responsibilities and accountability among a range of stakeholders and across jurisdictions. Like Uetake's (2013) organization style, which comprises organizations of farmers and agency participants, we found that organizational‐coalition collective action largely comprised government agencies and community members. Generally, we distinguished organizational‐coalition collective action from the others by virtue of the collaboration being largely between agencies and organizations, especially those with environmental mandates. The purpose of such organizational collaborations was to coordinate invasive species management programs and activities, pool resources, encourage consistent regulation and engagement (Higgins et al. 2007), or facilitate management at appropriate ecological scales (Thomsen & Caplow 2017).

This form of collective action involved organizations with a shared understanding of the socioecological system (Table 2). Possibly due to the scale at which this form of collective action operated, and the involvement of largely institutional actors, there was little focus on system productivity or predictability, resource unit mobility, graduated sanctions, or collective choice arrangements (Table 3). Instead, the focus was on functional aspects of these coalitions, such as the member roles, communication, decision making, and planning processes. Thus, success or failure of the coalitions tended to be judged in terms of interorganizational or individual characteristics rather than from a policy and institutional analysis perspective (Donaldson & Mudd 2010). In other words, studies about organizational coalition collective action generally stated or assumed that invasive species management requires collective action, although they included little or no discussion about which model, types, and scales of collective action were most appropriate, how these factors influenced conceptions of success, or how the collaboration related to existing bureaucratic and policy environments.

Future attempts to implement organizational coalition collective action should clearly articulate objectives, identify why and what type of collective action is required, and specify how success will be measured. It is important for members to not just share a desire to resolve an invasive species problem, but to clearly understand their responsibilities within the coalition and the extent of their jurisdiction. Success depends on establishing a clear definition of the invasive species problem from the outset as well as a management plan understood and agreed upon by all coalition members.

## Discussion

There is a developing, if nascent, interest in invasive species research that goes beyond individual managers and their practices to examine the relationships between various actors in invasive species management and the diverse configurations of collective action they form. Our typology, which extends similar efforts by Epanchin‐Niell et al. (2010), Marshall et al. (2016), and Uetake (2013), demonstrated the diversity of approaches to collective action. These approaches have reoriented invasive species policy, institutions, and management away from a narrow focus on educating and assisting individual managers and enforcing invasive species regulations on individual properties to more holistic, multiscalar, cross‐boundary, and collaborative efforts. In a broad sense, the works we reviewed made valuable contributions to conceptualizations of contemporary environmental management problems (including invasive species) that are defined by complexity and uncertainty and require inclusive, adaptive solutions (Woodford et al. 2016). Interorganizational and intersectoral collective action strategies have become more common, both as a policy tool for governments and as a means for organizations and resource managers to increase capacity, scope, and efficiency.

The articles reviewed provided insight for those seeking to enhance collective responses to invasive species. Local ownership (e.g., Lachapelle & McCool 2005) and capacity, social and financial, were critical for collective responses to endure, especially in instances where external support was high, but not assured in perpetuity. Financial capacity was important, but not sufficient for success. For example, collective efforts thrived when supported by normative beliefs among managers and stakeholders that invasive species ought to be controlled and that others were making investments to do so. Agencies or community leaders can elevate these normative beliefs with simple tools such as yard signs, public commitments, and participatory mapping (Niemiec et al. 2016) or existing communication networks. Across articles, collective responses were enhanced when stakeholders appreciated the cross‐boundary nature of the problem, were aware of the benefits that might arise from coordinated action, and were presented with achievable goals. Where organizations collaborated, clear problem definitions and roles of collaborators fostered more successful responses. Efforts to enhance these contextual factors are likely to boost collective invasive species control.

Although some of the invasive species collective action research reviewed was linked to broader CPR theory, in most cases these links remained opaque. Most articles discussed factors commonly associated with successful CPR collective action including norms and social capital, shared knowledge of the socioecological system, monitoring, and third‐party sanctioning (Tables 2 & 3). However, other factors identified as centrally or contextually important for effective CPR collective action, such as collective choice arrangements, low‐cost conflict‐resolution mechanisms, and collective‐choice rules, received less attention (Tables 2 & 3). Thus, there is significant scope for invasive species research to engage more substantively with CPR collective‐action theory and to clarify the type of collective‐action problems that exist with respect to invasive species and under what circumstances.

Invasive species management research could benefit from more deliberate engagement with PG theory and literature. Most reviewed articles uncritically engaged CPR theory in their discussions about the need for collective action in invasive species management. Our findings suggest that although some elements of CPR theory are relevant and applicable to collective control of invasive species, others have been confounded with PG‐specific characteristics and dynamics (i.e., establishing clear boundaries and number of users), and some may be incompatible (i.e., agencies or organizations monitoring and sanctioning individual behavior). More research is needed to explore and detail the aspects of CPR and PG theories that are complimentary when used to investigate collective responses to invasive species, which are contradictory, and, in cases of the latter, which is more fruitful. The relevance of these questions likely extends beyond invasive species control to other environmental resources.

Invasive species control represents a complex, interjurisdictional challenge that demands a range of collective actions linking diverse actors at various scales. This collective action requires a diversity of expertise that is functionally linked as an integrated system of management or mitigation. Thus, questions regarding the diverse forms of interactor collective action for invasive species management become highly germane. There is a need to better understand, connect, and model the conceptual foundations, forms, and actions of such collectives as a form of environmental network governance (Lubell et al. 2017).

Based on the results of our review, a key aim for the development of invasive species policy and governance should be to facilitate collective action between and among landowners, organizations, and government agencies to achieve management objectives at various scales. The legislative and policy foundations of invasive species management have remained largely unchanged, and there is a mismatch between their focus on individual‐level action and the complex, transboundary nature of invasive species and their management. There is considerable scope for future research on invasive species management to explore how policy and practice can more substantively draw on, be evaluated through, and contribute to rich bodies of existing theory and knowledge concerning environmental governance and collective action to empower effective responses to invasions and realize desired ecological outcomes.

## Supporting information

A list of the 21 publications that shaped the literature search (Appendix S1) and a summary of the invasive species and geographic foci of the reviewed articles (Appendix S2) are available online. The authors are solely responsible for the content and functionality of these materials. Queries (other than absence of the material) should be directed to the corresponding author.
