# Supplementary material for: Opportunities for better use of collective action theory in research and governance for invasive species management
Source: Conserv Biol. 2019 Jan 4;33(2):275–87. doi: 10.1111/cobi.13266 (PMC6850443; doi:10.1111/cobi.13266)
Supplement: Supplementary file 1 — A list of the 21 publications that shaped the literature search (Appendix S1) and a summary of the invasive species and geographic foci of the reviewed articles (Appendix S2) are available online. The authors are solely responsible for the content and functionality of these materials. Queries (other than absence of the material) should be directed to the corresponding author. [file COBI-33-275-s001.docx]

**Supporting Information 1: 21 articles that informed the design of the Scopus search terms**

Alexander J, Lee CA. 2010. Lessons learned from a decade of Sudden Oak Death in California: evaluating local management. Environmental Management **46**, 315-328.

Allen C, Horn C. 2009. Supporting collective action in pest management – aims and frameworks. Wellington, New Zealand: Landcare Research.

Ashcroft MB, Gollan JR, Batley M. 2012. Combining citizen science, bioclimatic envelope models and observed habitat preferences to determine the distribution of an inconspicuous, recently detected introduced bee (Halictus smaragdulus Vachal Hymenoptera: Halictidae) in Australia. Biological Invasions **14**:515-527.

Dangles O, Carpio FC, Villares M, Yumisaca F, Liger B, Rebaudo F, Silvain JF (2010) Community-based participatory research helps farmers and scientists to manage invasive pests in the Ecuadorian Andes. Ambio **39**:325-335.

De Lange WJ, Stafford WHL, Forsyth GG, Le Maitre DC. 2012. Incorporating stakeholder preferences in the selection of technologies for using invasive alien plants as a bio-energy feedstock: applkying the analytical hierarchy process. Journal of Environmental Management **99**:76-83.

Epanchin-Niell RS, Hufford MB, Asian CE, Sexton JP, Port JD, Waring TM. 2010. Controlling invasive species in complex social landscapes. Frontiers in Ecology and the Environment **8**:210-216.

Estevez RA, Anderson CB, Pizarro JC, Burgman MA. 2014. Clarifying values, risk perceptions, and attitudes to resolve or avoid social conflicts in invasive species management. Conservation Biology **29**:19-30.

Flora CB. 2007. Social Capital and Community Problem Solving: Combining Local and Scientific Knowledge to Fight Invasive Species. Available from: <https://vtechworks.lib.vt.edu/bitstream/handle/10919/67672/2873_FLORASocial_Capital_and_Community_Proble.doc?sequence=1> Accessed: 12 June 2016.

Ford-Thompson AES, Snell C, Saunders G, White PCL. 2012. Stakeholder participation in management of invasive vertebrates. Conservation Biology **26**:345-356.

Graham S. 2013. Three cooperative pathways to solving a collective weed management problem Australasian Journal of Environmental Manag **20**:116-129

Kadoya T, Ishii HS, Kikuchi R, Shin-ichi S, Washitani I. 2012. Biological conservation **142**:1011-1017.

McLeod LJ, Saunders GR. 2011. Can legislation improve the effectiveness of fox control in NSW? Australasian Journal of Environmental Management **18**:248-259.

Minato W, Curtis A, Allan C. 2010. Social Norms and Natural Resource Management in a Changing Rural Community. Journal of Environmental Policy & Planning **12**:381-403.

Niemiec RM, Ardoin NM, Wharton CB, Asner GP. 2016. Motivating residents to combat invasive species on private lands: social norms and community reciprocity. Ecology and Society **21**:30.

Ravnborg HM, Westermann O. 2002. Understanding interdependencies: stakeholder identification and negotiation for collective natural resource management. Agricultural Systems **73**:41-56.

Riethmuller J, McPhee S, McLean S, Ritchie D, Matthews J, Blackie D, Brown I, Box G. 2005. Facilitating the management of foxes on private land: are landholders interested? 13^th^ Australasian Vertebrate Pest Conference. Wellington, New Zealand. 2-6 May 2005.

Schyphers SS, Powers SP, Akins JL, Drymon JM, Martin CW, Schobernd ZH, Schfield PJ, Shipp RL, Switzer TS. 2014. The role of citizens in detecting and responding to a rapid marine invasion. Conservation Letters **8**:242-250.

Shine R, Doody JS. 2011. Invasive species control: understanding conflicts between researchers and the general community. Frontiers in Ecology and the Environment **9**:400-406.

Stokes KE, O’Neill KP, Montgomery WI, Dick JTA, Maggs CA, McDonald RA. 2005. The importance of stakeholder engagement in invasive species management: a cross-jurisdictional perspective in Ireland. Biodiversity and Conservation **15**:2829-2852.

Yung L, Chandler J, Haverhals M. 2015. Effective Weed Management, Collective Action, and Landownership Change in Western Montana. Invasive Plant Science and Management **8**:193-202.

Wakie TT, Laituri M, Evangelista PH. 2016. Assessing the distribution and impacts of Prosopis juliflora through participatory approaches. Applied Geography **66**:132-143.

**Supporting Information 2: Geographic distribution of invasive species collective action studies.**

| **Country** | **State** | **Articles** | **Invasive species** |
| --- | --- | --- | --- |
| Australia | National | Ford-Thompson *et al.* (2012) | Vertebrates |
|  | New South Wales | Berney *et al.* (2012)  Herbert (2013)  Graham (2013, 2014)  Klepeis *et al.* (2009)  Krueger (2016a, b)  Marshall *et al.* (2016)  McLeod and Saunders (2011) | Plants  Vertebrates |
|  | Queensland | Krueger (2016a, b) | Insects |
|  | Victoria | Graham (2013, 2014)  Mead (2016)  Minato (2010) | Plants |
| Bangladesh |  | Pretty and Ward (2001) | Insects, plants |
| Canada |  | Thomsen and Caplow (2016) | Plants |
| China |  | Pretty and Ward (2001) | Insects, plants |
| Colombia |  | Ravnborg and Westermann (2002) | Insects |
| India |  | Pretty and Ward (2001) | Insects, plants |
| Indonesia |  | Pretty and Ward (2001) | Insects, plants |
| Kazakhstan |  | Toleubayev *et al.* (2007) | Insects |
| Mexico |  | Ervin and Frisvold (2016) | Plants |
| Niger |  | De Groot (1995) | Insects |
| Philippines |  | Pretty and Ward (2001) | Insects, plants |
| Scotland |  | Bryce *et al.* (2011) | Vertebrates |
| Sri Lanka |  | Pretty and Ward (2001) | Insects, plants |
| United States | Alabama | Thomsen and Caplow (2016) | Plants |
|  | Arizona | Ayer (1997)  Hershdorfer *et al.* (2007) | Insects  Plants |
|  | California | Darin and Schoenig (2006)  Epanchin-Niell *et al.* (2010)  Ervin and Frisvold (2016) | Plants |
|  | Colorado | Hershdorfer *et al.* (2007)  Gunderson-Izurieta *et al.* (2008) | Plants |
|  | Delaware | Ervin and Frisvold (2016) | Plants |
|  | Florida | Higgins *et al.* (2007) | Plants |
|  | Hawaii | Niemiec *et al.* (2016) | Plants |
|  | Georgia | Ervin and Frisvold (2016)  Thomsen and Caplow (2016) | Plants |
|  | Missouri | Stallman and James (2015, 2016) | Insects |
|  | Montana | Yung *et al.* (2015) | Plants |
|  | Nevada | Donaldson and Mudd (2010) | Plants |
|  | New Mexico | Hershdorfer *et al.* (2007) | Plants |
|  | New York | Tette *et al.* (1987) | Insects |
|  | North Carolina | Thomsen and Caplow (2016) | Plants |
|  | South Carolina | Thomsen and Caplow (2016) | Plants |
|  | Tennessee | Thomsen and Caplow (2016) | Plants |
|  | Utah | Hershdorfer *et al.* (2007) | Plants |
|  | Virginia | Thomsen and Caplow (2016) | Plants |
| Vietnam |  | Pretty and Ward (2001) | Insects, plants |
